# Parenting styles and internet addiction in college students: self-esteem and self-control as mediators

**DOI:** 10.3389/fpsyg.2025.1555900

**Published:** 2025-10-31

**Authors:** Hongyu Gui, Qun Zhao, Yuchen Mao

**Affiliations:** ^1^School of Management Science and Engineering, Nanjing University of Information Science and Technology, Nanjing, Jiangsu, China; ^2^Research Institute for Environment and Health, School of Law and Public Administration, Nanjing University of Information Science and Technology, Nanjing, Jiangsu, China; ^3^Department of Statistics, University of South Carolina, Columbia, SC, United States

**Keywords:** parenting styles, internet addiction, self-control, self-esteem, college students

## Abstract

**Objectives:**

This study aimed to explore the effect of parental rearing styles on internet addiction among college students, specifically assessing the mediating roles of self-esteem and self-control in the influencing path.

**Methods:**

A cross-sectional questionnaire survey was conducted among college students in a city in eastern China in 2022. Chained mediation effect tests, employing Model 6 of PROCESS with SPSS (27.0), were utilized to statistically analyze the pathways of factors such as parenting styles, self-esteem, and self-control that influence Internet addiction.

**Results:**

Positive parenting styles served as protective factors against internet addiction, whereas negative parenting styles constituted potential risk factors. Self-esteem and self-control fully mediated the association between positive parenting styles and internet addiction. Conversely, they partially mediated the relationship between negative parenting styles and internet addiction.

**Conclusion:**

Interventions should attach importance to improving positive parenting styles and reducing negative parenting styles, and aim to cultivate self-esteem and strengthen self-control, which are essential to effectively address internet addiction in college students.

## 1 Introduction

As of 2021, global internet penetration surpassed 60%, with remarkably high rates of 93% in the U.S. and 67% in Asia. Asian people account for 54% of the global internet population (IWS, 2023). A significant portion of college students’ time is devoted to online gaming, social interaction, and information seeking, often hindering their ability to disengage (He et al., 2021; Zhang et al., 2023). While judicious internet use offers advantages, unchecked usage frequently creates challenges and may even result in internet addiction (Liu et al., 2017). Moreover, the nomenclature surrounding excessive and detrimental internet use remains contested and can be referred to as internet addiction, pathological internet use, internet dependence, problematic internet use, or excessive internet use (Yang and Kim, 2021; Cash et al., 2012; Moretta and Buodo, 2020). This study adopts the term “internet addiction”—prevalent in China—to denote an excessive preoccupation with the internet (Guo et al., 2024).

Internet addiction is prevalent among middle school, high school, and college students worldwide (Wallace, 2014). Students who experience internet addiction may develop mental health problems, risky emotional regulation, and social anxieties (Yung et al., 2015), among other challenges. Internet addiction can result in a range of negative effects for students, including poor concentration and lower academic performance (Dol, 2016), mood and anxiety disorders (Spada, 2014), high levels of aggression (Ko et al., 2009), personality disorders (Jiang and Leung, 2012), and impulsivity (De Berardis et al., 2009). Therefore, understanding the factors that contribute to internet addiction is crucial. Research indicates a strong association between parenting styles and internet addiction in children, adolescents, and college students (Tang et al., 2018; Karaer and Akdemir, 2019; Agbaria and Bdier, 2022; Li et al., 2018). While Chinese parents often demonstrate characteristics consistent with authoritarian parenting, their behavioral patterns may reflect cultural norms, values, and beliefs that diverge from the Western conceptualization of authoritarianism, as described by Floros et al. (2013). In collectivist cultures like China, parenting practices often emphasize obedience and academic pressure. Behaviors labeled “authoritarian” in the West may be seen as caring or supportive in the East Asian context (Baumrind, 1991; Chao, 1994). Therefore, assessing the effects of both supportive and detrimental parenting practices among Chinese parents on internet addiction in college students holds considerable practical importance.

### 1.1 Variables and objectives

#### 1.1.1 Parenting styles

According to typical patterns of parental attitudes and emotional support offered to children, parenting styles are recognized as influential determinants of children’s emotional and behavioral development (Safdar and Zahrah, 2016), and reflect consistent patterns of parental attitudes, responsiveness, and disciplinary strategies. Parental styles are classified into four primary categories: neglectful, permissive, authoritarian, and authoritative (Chao, 1994). Drawing on Safdar and Zahrah (2016), these styles are broadly characterized as either positive (authoritative types) or negative (permissive, neglectful and authoritarian types) in their influence.

Research consistently indicates that parenting style is a strong predictor of internet addiction among college students (Huang et al., 2010; Najia and Sara, 2024; Sharabany et al., 2008; Siomos et al., 2012; Valcke et al., 2010). Positive parenting practices, such as emotional warmth, appear to protect against internet addiction, whereas negative parenting practices, such as rejection and overprotection, represent possible risk factors for their development (Guo et al., 2024). When a warm family environment is lacking, college students may compensate by seeking comfort in online activities, a behavior that can escalate into internet addiction (Zhang et al., 2019). In the East Asian cultural context, parenting styles exhibit distinct expressions and impacts compared to Western societies. Research indicates that while authoritarian parenting is commonly categorized as a negative style, it may not universally yield adverse outcomes within collectivist cultures, where societal norms emphasize obedience and hierarchical family structures (Chao, 1994; Rudy and Grusec, 2006). Considering that the effects of authoritative and authoritarian parenting styles can vary across cultural environments, parental warmth was chosen as a representative positive parenting style for the analysis in this study.

#### 1.1.2 Self-esteem and self-control

Self-esteem, defined as an individual’s overall sense of worth and holistic appraisal of personal value (Brailovskaia and Margraf, 2020), has been found to correlate negatively with internet addiction (Dai et al., 2024; Kumar and Mondal, 2016). Adolescents and students with low self-esteem may engage in excessive internet use to compensate for their inadequacies, alleviate social anxiety, or reduce emotional distress. This behavior reflects attempts to seek online validation and reduce discomfort, contributing to internet dependency (Shi et al., 2017; Yücens and Üzer, 2018). Such compensatory behavior can become habitual, ultimately fostering internet addiction (Du et al., 2024; Niemz et al., 2005; Griffiths, 2000).

Research suggests a crucial relationship between college students’ self-esteem and their parents’ child-rearing practices. Empirical evidence indicates that children reared in environments characterized by emotional warmth and parental validation tend to cultivate robust self-worth, whereas exposure to chronic criticism or neglect heightens vulnerability to low self-esteem (Milevsky et al., 2007). Moreover, self-esteem is positively related to self-control, suggesting a cascading effect whereby parenting influences self-esteem, which then affects self-regulation capacity (Tangney et al., 2004).

Self-control, the conscious regulation of impulses, habits, and automatic responses in service of long-term goals (Tangney et al., 2004; Carver and Scheier, 2002), has been found to correlate inversely with internet addiction in middle school and college students (Bagatarhan et al., 2023; Zhang et al., 2022; Li et al., 2021; Du and Zhang, 2022; Agbaria and Bdier, 2021; Li et al., 2021). Adolescents and college students with poor self-control are more likely to engage in impulsive online behaviors, prioritize immediate gratification, and struggle to moderate their internet use, especially in the absence of external supervision (Bagatarhan et al., 2023; Zhang et al., 2022; Li J. J. et al., 2021; Du and Zhang, 2022; Agbaria and Bdier, 2021; Li S. Q. et al., 2021; Tang et al., 2015; Stein and Witkiewitz, 2019; Hameed and Irfan, 2021). Parenting styles play a crucial role in shaping the development of self-control. Positive parenting cultivates self-regulatory abilities through consistent behavioral modeling, clear boundary setting, and autonomy support (Eisenberg et al., 2005). Conversely, negative parenting, particularly permissive or authoritarian approaches, is significantly correlated with heightened impulsivity and self-regulatory deficits (Lengua, 2006).

Besides, gender differences in internet addiction have been increasingly observed. Male students show higher levels of internet addiction than females, due to greater involvement in online gaming and risk-taking behaviors (Günaydin, 2021; Mehroof and Griffiths, 2010). However, females may be more susceptible to social media-related addiction. Studies suggest that parenting styles’ impact on internet addiction differs by gender, with maternal warmth showing stronger associations with reduced addiction in female students, while paternal control may have greater effect on males (Yao et al., 2013; Shek et al., 2018). These findings highlight the need to examine potential gender-based moderating effects on the pathways linking parenting styles to internet addiction (Blase et al., 2021; Chen et al., 2019; Duckworth et al., 2019; Ebeid et al., 2019; Kiel and Maack, 2012; Liu et al., 2021; Oliva et al., 2019; Troll et al., 2021).

### 1.2 Objectives

**Figure 1:**
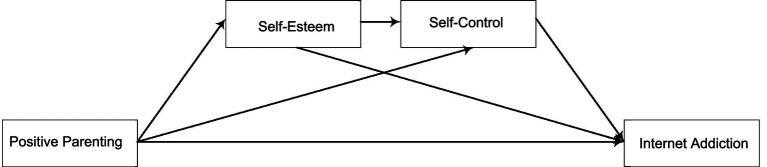
Conceptual model.

Previous studies have demonstrated the mediating effects of both self-esteem and self-control on the relationship between parenting style and Internet addiction (Dai et al., 2024; Bagatarhan et al., 2023). However, research exploring the mediating mechanisms of these factors among college students remains scarce. A thorough understanding of these mediating factors and how they function in the relationship between diverse parenting styles and Internet addiction among college students is essential, as such insights can contribute significantly to preserving the mental health of this population. In addition, to the best of our knowledge, research on the roles of self-esteem and self-control in internet addiction among college students, and particularly on their mediating influence on the association between parenting styles and Internet addiction, is generally lacking. Therefore, this study aims:

- to understand the relationship between different parenting styles and internet addiction in the undergraduate student population.
- to explore how self-esteem and self-regulation mediate the relationship between parenting approaches and internet addiction, and to compare differences in these mediating effects across different parenting styles (see Figure 1).

## 2 Methods

### 2.1 Subjects of study

This study draws upon data from a 2022 cross-sectional survey administered in a major, economically prosperous city in eastern China. Participants were randomly sampled from four colleges in a single university. Senior students, primarily engaged in off-campus internships, were excluded from the sample. Questionnaires were administered in classrooms after scheduled classes. From the 2,190 questionnaires collected, 2,065 were considered valid after excluding those with missing data, resulting in a valid response rate of 94.3%.

### 2.2 Research variables

#### 2.2.1 Positive parenting styles

This construct was assessed through three items, “Parental providers allow me to be unique,” “I can feel loved by my parents/providers,” and “When things are not going my way, my parental providers always encourage and help me.” Each item employed a 4-point scale ranging from 1 (never) to 4 (often), with higher scores indicating higher levels of caring parental behavior. These items were drawn from the Egna Minnen av Barndoms Uppfostran (EMBU) (Arrindell et al., 1998). For analytical purposes, a composite variable was constructed by averaging the responses to these three items. Reliability analysis derived a Cronbach’s alpha of 0.763. Validity was assessed through KMO sampling adequacy, which was 0.681 (*p* < 0.0001).

#### 2.2.2 Negative parenting styles

This construct was assessed utilizing items reflecting controlling, neglectful, and permissive parenting styles. Example items include: “My parents interfered with everything I did when I was growing up,” “My parents favored me when I got into trouble,” and “My parents have no time to take care of me.” Each item employed a 4-point Likert scale ranging from 1 (never) to 4 (often), with higher scores signifying greater parental neglect. These items were drawn from the Egna Minnen av Barndoms Uppfostran (EMBU) (Arrindell et al., 1998). The mean score was calculated for analysis. Reliability: Cronbach’s Alpha = 0.779; Validity: KMO Sampling Suitability Measure = 0.833, Significance <0.0001.

#### 2.2.3 Self-esteem levels

This construct was measured utilizing items such as “I feel that I am as useful as anyone else,” “I feel that I have many advantages,” and “I can do things as well as most people.” Responses were recorded on a 4-point scale ranging from 1 (very poor) to 4 (very well), with higher scores indicating greater self-esteem among college students. This scale is adapted from the Rosenberg Self-Esteem Scale developed by American psychologist Rosenberg (1989), a widely utilized measure of personal self-confidence. A mean score was calculated for analysis. Reliability: Cronbach’s Alpha = 0.916; Validity: KMO Sampling Suitability Scale = 0.893, Significance <0.0001.

#### 2.2.4 Self-control levels

This construct was evaluated with items such as “I often cannot resist temptation,” “It is difficult for me to get rid of bad habits,” and “I am lazy.” Each item utilized a 5-point scale, ranging from 1 (strongly agree) to 5 (completely disagree). Higher scores corresponded to greater self-control among college students. The self-control items used in this study were compiled from the standardized College Students’ Self-Control Scale (Tan and Guo, 2008). A mean score was computed for analysis. Reliability: Cronbach’s Alpha = 0.885; Validity: KMO Measure of Sampling Adequacy = 0.919, Significance <0.0001.

#### 2.2.5 Internet addiction (10-item internet addiction test: internet addiction test—10, IAT-10)

This construct was evaluated with items such as, “Are you too concerned about the Internet,” “Do you feel the need to increase your internet time to feel satisfied,” and “Is it difficult for you to reduce or control your use of the Internet.” Each item was scored dichotomously, either 1 (yes) or 0 (no). This study utilized the 10-item Internet Addiction Scale developed by Brand et al. (2014), which comprises 10 Internet addiction symptoms. Participants responded “yes,” scored 1 or “no,” scored 0 to items reflecting their internet use over the past year, with higher scores indicating greater internet addiction. Reliability: Cronbach’s Alpha = 0.885; Validity: KMO Measure of Sampling Adequacy = 0.899, significance <0.0001.

Control variables: gender was used as a control variable.

### 2.3 Methodology

SPSS version 22.0 was employed for descriptive statistics and correlation analysis. Following Hayes’s procedure (Bolin, 2014), the PROCESS statement was implemented for chained mediation modeling. Moderated mediation models (Model 6, Chained Mediated Effects Model) in PROCESS tested direct and indirect effects. All study variables were *Z*-score standardized prior to analysis to enhance interpretability. Indirect effects were tested utilizing bias-corrected percentile bootstrapping with 5,000 resamples. The conditional indirect effect, or detection interaction, achieved statistical significance, as evidenced by the 95% confidence intervals, not including zero.

Confirmatory Factor Analysis (CFA) was conducted using Mplus 8.3 to validate the measurement model. The results of confirmatory factor analysis (CFA) showed that the model demonstrated good overall fit: *χ*^2^ = 2535.632 (df = 517, *p* < 0.001), *χ*^2^/df = 4.90, CFI = 0.931, TLI = 0.925, RMSEA = 0.043 (90% CI: 0.042–0.045), and SRMR = 0.033. All indices met or exceeded recommended thresholds (Hu and Bentler, 1999), confirming the structural validity of the theoretical constructs.

College students were selected due of their high exposure to internet use and increased risk of addiction during a critical stage of psychological development transitioning from adolescence to adulthood. Additionally, this group often experiences academic stress and greater autonomy, making it relevant for examining the influence of parenting on self-esteem and self-control.

## 3 Results

### 3.1 Demographics

The demographic information of the study population was as follows: gender (47.2% for boys and 52.8% for girls) and an average age of approximately 19 years. Scores for positive parenting (boys 3.49, girls 3.64, *p* < 0.0001), negative parenting (boys 1.92, girls 1.79, *p* < 0.0001), self-esteem levels (boys 3.03, girls 3.09, *p* < 0.0472), self-control levels (boys 3.30, girls 3.11, *p* < 0.0001), and levels of Internet addiction (boys 0.32, girls 0.35, *p* < 0.0001) were recorded. The analysis indicated no statistically significant difference in age between male and female college students; however, statistically significant differences were observed across other research variables. As a result, gender was included as a control variable in the mediation model to account for its potential confounding effects (see Table 1).

**Table 1:** Bivariate comparison of socio-demographic characteristics of college students.

Variable	Gender	Subjects	Mean	Standard deviation	Significance level (two-tailed)*
Age	Male	974	19.02	1.078	0.6280
	Female	1,088	19.00	0.951	
Positive parenting	Male	974	3.49	0.623	<0.0001
	Female	1,091	3.64	0.513	
Negative parenting	Male	974	1.92	0.490	<0.0001
	Female	1,091	1.79	0.469	
Self-esteem	Male	974	3.03	0.638	0.0472
	Female	1,091	3.09	0.556	
Self-control	Male	974	3.30	0.852	<0.0001
	Female	1,091	3.11	0.816	
Internet addiction	Male	974	0.32	0.286	0.0139
	Female	1,091	0.35	0.282	

*Statistical comparisons were made at the 0.05 level (two-tailed) using the *t*-test.

### 3.2 Correlation test

Table 2 presents the correlations between the variables. As presented in Table 2, these correlations align with expectations. Specifically, Internet addiction among college students demonstrates a negative correlation with positive parenting styles and a positive correlation with negative parenting styles. Besides, Internet addiction among college students is negatively correlated with both self-esteem and self-control levels. These findings suggest that negative parenting styles may constitute a risk factor for Internet addiction among college students, whereas positive parenting styles, self-esteem, and self-control represent protective factors.

**Table 2:** Correlation of variables.

Variable	Positive parenting	Negative parenting	Self-esteem	Self-control
Negative parenting	−0.352^**^			
Self-esteem	0.295^**^	−0.227^**^		
Self-control	0.162^**^	−0.198^**^	0.292^**^	
Internet addiction	−0.111^**^	0.185^**^	−0.260^**^	−0.492^**^

**Statistical comparisons were made at the 0.01 level (two-tailed) using Pearson’s correlation coefficient.

### 3.3 Tests for chained mediation effects of dependency patterns

Applying gender as a control variable, positive parenting style score as the independent variable, and self-esteem (M1) and self-control (M2) as mediating variables, we analyzed the effect on Internet addiction. The results showed that positive parenting styles significantly predicted reduced internet addiction through indirect pathways [*β* = −0.119, SE = 0.013, 95% CI (−0.143, −0.094)], while the direct effect was not significant [*β* = −0.001, SE = 0.020, 95% CI (−0.041, 0.038)]. This suggests a full mediation model for the study.

Three indirect pathways were identified: (IWS, 2023) through self-esteem alone [*β* = −0.037, SE = 0.007, 95% CI (−0.052, −0.023)], (He et al., 2021) through self-control alone [*β* = −0.045, SE = 0.011, 95% CI (−0.066, −0.024)], and (Zhang et al., 2023) through the sequential path from self-esteem to self-control [*β* = −0.036, SE = 0.005, 95% CI (−0.046, −0.027)].

Although the individual effect sizes were modest, they were statistically robust, with narrow confidence intervals and low standard errors, indicating stable estimates. From a practical standpoint, these findings suggest that positive parenting reduces internet addiction by enhancing psychological resilience, particularly by fostering higher self-esteem and self-control in college students. These results are presented in Table 3.

**Table 3:** Mediating effects and confidence intervals for positive parenting styles.

Name	Path	Point estimates	SE	95%CI lower limit	95%CI upper limit
Direct effect	Positive parenting → internet addiction	−0.001	0.020	−0.041	0.038
Indirect effect		−0.119	0.013	−0.143	−0.094
Indirect effect 1	Positive parenting → Self-esteem → internet addiction	−0.037	0.007	−0.052	−0.023
Indirect effect 2	Positive parenting → self-control → internet addiction	−0.045	0.011	−0.066	−0.024
Indirect effect 3	Positive parenting → self-esteem → self-control → internet addiction	−0.036	0.005	−0.046	−0.027

In contrast, negative parenting styles had a significant total effect on internet addiction (*β* = 0.196, SE = 0.017), including both a direct effect [*β* = 0.075, SE = 0.020, 95% CI (0.036, 0.114)] and a significant indirect effect through self-esteem and self-control [*β* = 0.121, SE = 0.014, 95% CI (0.095, 0.149)]. This finding indicates partial mediation, with indirect paths accounting for approximately 61.7% of the total effect.

The strongest indirect effect was via self-control alone [*β* = 0.069, SE = 0.012, 95% CI (0.047, 0.093)], followed by the effect through self-esteem (*β* = 0.026) and the sequential pathway from self-esteem to self-control (*β* = 0.026). These findings underscore that negative parenting increases the risk of internet addiction, particularly by impairing the development of self-regulation and psychological self-worth (see Table 4).

**Table 4:** Mediating effects and confidence intervals for negative parenting.

Name	Path	Point estimates	SE	95%CI lower limit	95%CI upper limit
Direct effect	Positive parenting → internet addiction	0.075	0.020	0.036	0.114
Indirect effect		0.121	0.014	0.095	0.149
Indirect effect 1	Positive parenting → self-esteem → internet addiction	0.026	0.006	0.015	0.038
Indirect effect 2	Positive parenting → self-control → internet addiction	0.069	0.012	0.047	0.093
Indirect effect 3	Positive parenting → self-esteem → self-control → internet addiction	0.026	0.004	0.019	0.035

Taken together, these results suggest that both self-esteem and self-control are critical psychological mechanisms that mediate the influence of parenting styles on problematic internet use. These mediators not only offer theoretical insights but also point to potential intervention targets for reducing internet addiction risk among college students. The robustness of these findings is supported by the large sample size (*N* > 1,000), which ensures high statistical power and precise estimates, as evidenced by the narrow confidence intervals.

## 4 Discussion

This study explored the relationship among parenting styles, self-esteem, self-control, and Internet addiction. The findings shed light on potential mechanisms underlying the association between parenting styles and Internet addiction and offer implications for the development of effective intervention strategies to address Internet addiction.

### 4.1 Relationship between parenting styles and internet addiction

Consistent with existing research, this study demonstrated that positive parenting styles (characterized by warmth, responsiveness, and support) are associated with lower levels of internet addiction in college students, while negative parenting styles (e.g., neglectful, permissive parenting) demonstrated a positive correlation. Specifically, neglectful or permissive parenting, often represented by a lack of supervision regarding problematic behaviors and a greater tendency to fulfill children’s needs and desires (Sharabany et al., 2008), has been strongly associated with Internet addiction (Huang et al., 2010). In addition, authoritarian parenting, which demands obedience and conformity from children, can contribute to a poor self-concept, undesirable behaviors, and a pursuit of alternative social interactions through the Internet, thereby increasing the likelihood of Internet addiction (Najia and Sara, 2024). These findings align with existing studies suggesting that authoritarian parenting may hinder a child’s autonomy and foster reliance on external sources, such as the internet, to fulfill unmet psychological needs (Najia and Sara, 2024; Sun, 2023; Chou et al., 2016; Liu et al., 2024).

The Internet Satisfaction of Psychological Needs Theory hypothesizes that when university students’ psychological needs remain unfulfilled in their offline lives, they may turn to online avenues, such as the Internet, to find gratification. Should the Internet successfully fulfill these needs, it could encourage more frequent Internet use, potentially leading to addictive online behaviors (Liu et al., 2016). These findings underscore the importance of parenting styles in shaping internet use patterns among college students in collectivist cultures such as China, where parental influence remains significant into young adulthood (Chao, 1994).

### 4.2 Mediating role of self-esteem level

Self-esteem significantly mediated the relationship between parenting styles and internet addiction. Positive parenting supports autonomy and self-worth, fostering resilience to addictive tendencies. Conversely, authoritarian or controlling parenting can undermine self-esteem by emphasizing obedience over personal agency (Patock-Peckham and Morgan-Lopez, 2009). Kumar’s research indicates that college students with lower self-esteem exhibit higher rates of Internet addiction, while those with higher self-esteem demonstrate a lower tendency toward Internet addiction, indicating a significant negative correlation between these two variables (Kumar and Mondal, 2016). This finding may be explained by the tendency of individuals with low self-esteem to question their capabilities and utilize the Internet as a means of escaping reality and seeking psychological solace (Toma, 2022).

### 4.3 Mediating role of self-control

This study reaffirms the importance of self-control as a key predictor of Internet addiction among college students (Jiang and Zhao, 2016; Wei et al., 2020). In line with self-control theory, individuals with poor self-control are more inclined toward immediate gratification and are thus at a higher risk of developing maladaptive behaviors, including excessive internet use (Zhang and Wang, 2023; Gottfredson and Hirschi, 1990). Parenting styles play a critical role in shaping self-regulatory capacities; positive parenting fosters emotional regulation and delayed gratification, whereas negative parenting diminishes a child’s capacity to self-regulate. These findings reinforce the importance of self-control as a protective factor and intervention target for adolescents (Tangney et al., 2004; Kim et al., 2017; Chapple, 2005).

### 4.4 Chain mediation of self-esteem and self-control and differences

In contrast to previous research, this study identified the mediating effects of various parenting styles on Internet addictive behaviors through the mechanisms of self-esteem and self-control. Moreover, we observed different pathways of influence for positive and negative parenting styles.

Self-esteem and self-control fully mediated the association between positive parenting styles and internet addiction, while they only partially mediated the association between negative parenting styles and internet addiction. Theoretically, self-esteem affects how individuals react to interpersonal feedback, allowing them to undertake corrective measures (Brown, 2010; Ford and Collins, 2013). The theory of threatened egocentrism hypothesizes that individuals with higher self-esteem will modify their behavior to preserve initial positive evaluations (Baumeister et al., 1996). This capacity for emotional regulation is governed by self-control. Self-control influences this capacity for emotional regulation (Campbell and Riggs, 2015). In addition, individuals with strong self-control may demonstrate higher self-esteem and a more robust sense of self (Laible et al., 2004). Therefore, we hypothesize that positive parenting styles facilitate the development of healthy self-esteem and increased self-control in college students, enabling them to adapt to college life and lessening the degree of internet addiction without direct parental intervention, whereas college students raised with negative parenting styles present higher levels of internet addiction, likely attributable to lower levels of self-esteem and a hindered capacity to cultivate greater self-control, driven by a psychological inclination to avoid reality. We contend that the cohort of college students who experienced positive parenting styles has cultivated comparatively greater psychological resilience, thereby reducing the role of parental influence on the prevention of internet addiction. Simultaneously, lower levels of self-esteem and self-control may render these students more vulnerable to the lasting effects of negative parenting styles, directly contributing to the exacerbation of internet addiction.

## 5 Limitations and future research

Firstly, relying on self-reported data introduces the potential for social desirability and recall biases. Secondly, the cross-sectional nature of this study precludes causal interpretations. Future longitudinal designs should track parenting style transitions during university (e.g., increased autonomy granting) and their lagged effects on internet addiction through self-regulatory mechanisms. Thirdly, while the measurement instruments have been verified for Chinese college students, in the East Asian context, strict parenting may reflect care and responsibility rather than control, potentially altering its psychological impact (Chao, 1994). This may explain why negative parenting does not always lead to poor outcomes in collectivist cultures. Future analyses should explore cultural differences and account for potential response bias.

Notwithstanding these limitations, this research contributes important insights to the existing literature on internet addiction among college students, indicating that parental upbringing significantly affects internet addiction through self-esteem and self-regulation. The findings support a multidimensional framework for addressing internet addiction, suggesting that interventions should be both psychological (focusing on internal traits such as self-esteem and self-control) and familial (encouraging constructive parenting).

## 6 Conclusion and practical implications

This study underscores the role of parenting styles in internet addiction, mediated by self-esteem and self-control, with the cultural context shaping these pathways. These findings have significant implications for intervention and prevention strategies. Universities can implement programs to enhance self-esteem and self-control, such as cognitive-behavioral workshops or mindfulness training, to reduce internet addiction risk. Parents should be educated on adopting positive parenting practices, such as warmth and autonomy support, through community-based workshops. Policymakers should fund campus mental health initiatives targeting students from negative parenting backgrounds to address their unique vulnerabilities. Theoretically, this study extends Internet Satisfaction of Psychological Needs Theory by integrating self-esteem and self-control as mediators in a collectivist context, offering a nuanced model of internet addiction. The chained mediation model highlights the interplay of psychological mechanisms, thereby advancing research on parenting and addiction.

## Data Availability

The raw data supporting the conclusions of this article will be made available by the authors, without undue reservation.
